# Ovarian Function, Hormonal, Metabolic, Oxidative Stress, and Gene Expression Biomarker Responses of Holstein Heifers Under Heat Stress Conditions to a Reduced Dose of Nanofabricated GnRH Analog Within the Ovsynch Protocol

**DOI:** 10.3390/ani16142163

**Published:** 2026-07-12

**Authors:** Eman M. Hassanein, Ottó Szenci, Rezk S. Ghallab, Ahmed A. Saleh, Mariam S. Abdelfattah, Ebrahim F. Zayed, Mohamed F. Kilany, Zoltán Szelényi, Abdelghany A. El-Shereif

**Affiliations:** 1 Department of Obstetrics and Farm Animal Medicine Clinic, University of Veterinary Medicine, István u. 2, H-1078 Budapest, Hungary; em.mostafa@alexu.edu.eg (E.M.H.); szelenyi.zoltan@univet.hu (Z.S.); 2 Animal and Fish Production Department, Faculty of Agriculture (Al-Shatby), Alexandria University, Alexandria 11865, Egypt; elemlak1339@gmail.com (A.A.S.); mariam.said017@alexu.edu.eg (M.S.A.); ebrahimzayed@gmail.com (E.F.Z.); abdelghany.awad@alexu.edu.eg (A.A.E.-S.); 3 Theriogenology Department, Faculty of Veterinary Medicine, Matrouh University, Matrouh 51512, Egypt; rezkgallab@gmail.com; 4 Faculty of Veterinary Medicine, University of Sadat City, Sadat City 6011007, Menoufia Governorate, Egypt; mohamedfawzykelany96@gmail.com

**Keywords:** drug delivery, chitosan nanoparticles, follicular dynamics, luteal function, antioxidant status, gene expression, ovulation synchronization

## Abstract

This study investigated the efficacy of a modified Ovsynch (MOVS) protocol incorporating a 50% reduced dose of a gonadotropin-releasing hormone analog (GnRH_a_) conjugated with chitosan nanoparticles. Thirty-two Holstein heifers under heat-stress (HS) conditions were randomly assigned to either a standard Ovsynch (SOVS) or MOVS protocol (n = 16/group). Ovarian dynamics were monitored across the protocol days (D0, D3, D7, D9, D14, and D19). Hormonal profiles, metabolic and oxidative markers, and mRNA expressions of folliculogenesis- and apoptosis-related genes were evaluated. Both protocols improved follicular recruitment (*p* > 0.05). However, the MOVS significantly enhanced the dominant follicle (DF) diameter on D9 (*p* < 0.001). On D14 and D19, CL diameters were larger in the MOVS group (*p* < 0.001). These findings were associated with significantly high estradiol (E_2_) concentrations on D9 (*p* < 0.05) and progesterone (P_4_) concentrations on D19 (*p* < 0.05). Metabolic and oxidative stress markers were increased in MOVS-treated heifers, while antioxidant markers were also improved (*p* < 0.05). At the molecular level, the MOVS numerically upregulated the expression of folliculogenesis-related genes (*BMP15*, *GDF9*, *LHR*, *FSHR*, and *IGF-1R*) and an anti-apoptotic gene (*BCL2*) in peripheral blood (*p* > 0.05). In conclusion, the MOVS protocol achieved a 50% reduction in GnRHa dose while improving ovarian, endocrine, and reproductive physiological indicators, suggesting a promising strategy for optimizing synchronization in heifers under HS conditions.

## Simple Summary

This current study examined the effectiveness of a reduced dose of a gonadotropin-releasing hormone analog (GnRHa) conjugated to chitosan nanoparticles as nanocarriers within the Ovsynch protocol in Holstein heifers under heat-stress (HS) conditions. Our findings highlight that nanofabricated GnRH_a_ enhanced ovarian activity, endocrine profiles, metabolic response, and antioxidant status. Additionally, it was associated with numerical increases in the circulating expression of folliculogenesis-related genes and an anti-apoptotic gene compared with the standard Ovsynch protocol. These results indicate enhanced physiological readiness for reproduction and suggest a promising strategy to optimize synchronization in heifers under HS conditions. Moreover, this strategy enabled a 50% reduction in the hormonal dose while preserving or even enhancing its effectiveness. Overall, this study demonstrates the potential of incorporating nanotechnology into reproductive synchronization protocols in heifers subjected to HS conditions, offering a promising prospect for enhancing physiological responses associated with reproductive performance.

## 1. Introduction

Maximizing the profitability of dairy herds necessitates effective reproductive management. In dairy heifers, implementing appropriate reproductive management strategies is essential to sustain herd replacement and enhance reproductive performance by ensuring timely breeding and proper regulation of ovarian activity [1]. Reproductive performance in dairy herds is affected by several factors, including health status, nutrition, management practices, and environmental factors [2]. Among these, heat stress (HS) is considered a critical environmental factor limiting reproductive efficiency in dairy herds [3,4,5].

The severity of HS is commonly quantified using the temperature–humidity index (THI), which integrates ambient temperature (AT) and relative humidity (RH) into a composite value [4,6,7]. However, THI thresholds may vary depending on climate conditions, production system, and geographic region [8]. In tropical and subtropical regions, dairy cattle are considered heat-stressed when the THI exceeds 72 [6,9,10]. Numerous studies have demonstrated a negative association between a high THI and reproductive performance in dairy cows and heifers [5,9,11,12,13,14,15]. Exposure to high AT increases core body temperature and energy expenditure while adversely affecting feed intake, a physiological attempt to attenuate metabolic heat production that ultimately compromises reproductive performance [5,9,16,17,18].

Although most studies have focused on lactating cows, dairy heifers are equally vulnerable to HS [19,20,21]. Exposure to HS impairs fertility in heifers mainly through detrimental effects on ovarian function and fertility outcomes [20]. HS affects reproduction through both direct and indirect pathways [22]. Directly, HS activates the hypothalamic–pituitary–adrenal (HPA) axis, increasing cortisol secretion as a normal response to stress, thereby affecting the hypothalamic–pituitary–ovarian (HPO) axis, interfering with folliculogenesis and steroidogenesis, and reducing follicular competence and oocyte quality, ultimately impairing ovarian activity [12,19,23,24,25,26]. Additionally, HS adversely affects corpus luteum (CL) formation and decreases progesterone (P_4_) synthesis, as luteinized granulosa cells (GCs) and theca cells (TCs) from follicles exposed to HS produce lower P_4_ concentrations than those from cooler seasons, ultimately impairing pregnancy (PR) maintenance [27].

At the cellular level, HS induces oxidative stress through excessive accumulation of reactive oxygen species (ROS) and impairs antioxidant defense systems [28,29]. High levels of ROS disrupt cellular redox balance, damage proteins, lipids, DNA, and mitochondria, and reduce ATP production, ultimately compromising oocyte and GC function [30,31,32]. Concurrently, reduced antioxidant capacity exacerbates oxidative damage, while antioxidant enzymes such as superoxide dismutase (SOD) and catalase (CAT) can partially counteract the accumulation of ROS. Consequently, the imbalance between ROS production and intracellular antioxidant capacity adversely affects oocyte developmental competence [33]. Additionally, excessive ROS generation activates apoptotic pathways by altering the expression of proapoptotic genes relative to anti-apoptotic genes [34]. HS compromises GCs’ viability by activating apoptotic pathways, including caspase-3 activation and disruption of the balance between the proapoptotic BCL2-associated X protein (BAX) and the anti-apoptotic B-cell lymphoma 2 (BCL2) [35]. This imbalance reduces the expression of steroidogenic genes, suppresses aromatase activity, and consequently decreases estradiol (E_2_) synthesis in GCs, ultimately compromising follicular function under HS conditions [29,31,36]. Collectively, these alterations impair follicular development, oocyte quality, steroidogenesis, and reproductive performance under HS.

Furthermore, HS adversely affects several molecular pathways involved in folliculogenesis and steroidogenesis, compromising ovarian activity and follicular function and reducing fertility [26]. For instance, HS has been reported to downregulate the expression of folliculogenesis-related genes, including growth differentiation factor 9 (*GDF9*) and bone morphogenetic protein (*BMP15*) [36]. It impairs the expression of fundamental steroidogenic genes, including the steroidogenic acute regulatory protein (*STAR*) and the cytochrome P450 enzymes CYP11A1, CYP17A1, and CYP19A1 (*aromatase*) [26]. It also affects the expression of key gonadotropin receptors, including follicle-stimulating hormone receptor (*FSHR*) and luteinizing hormone receptor (*LHR*), which are essential for regulating follicular development, steroidogenesis, ovulation, and CL development [37,38]. To our knowledge, limited studies have evaluated *FSHR* and *LHR* expression in cattle under HS conditions. However, in goats, HS has been shown to alter the expression of both *FSHR* and *LHR*, thereby impairing follicular dynamics, steroidogenic activity, and ovarian function [39].

HS also has an indirect impact on reproductive performance by decreasing feed and dry matter intake. This reduces key metabolic parameters, such as insulin, insulin-like growth factor-1 (IGF-1), and glucose, which are necessary for normal follicle growth, oocyte quality, and ovulation [22,40,41].

Estrus expression, intensity, and detection are adversely affected by HS; therefore, reproductive management strategies that improve synchronization and breeding efficiency have become increasingly important [22,28,42,43,44]. Timed artificial insemination (TAI) protocols are beneficial because they eliminate the need for estrus detection and enable precise synchronization of ovulation with insemination [45]. Different synchronization protocols have demonstrated beneficial effects on reproductive performance during the summer season [46]. Among these protocols, the Ovsynch protocol (OVS), which relies on the sequential administration of GnRH and prostaglandin F_2α_ (PGF_2α_), is among the most widely used synchronization protocols in dairy cattle [45,47]. Despite its effectiveness, conventional GnRH administration generally has several limitations, particularly in summer conditions, due to rapid degradation and a short biological half-life [48]. These limitations may reduce luteinizing hormone (LH) release and impair synchronization efficiency [49]. Therefore, alternative approaches that improve hormonal stability and delivery efficiency are required.

Nanobiotechnology has recently emerged as a promising approach to improve reproductive management strategies through controlled hormonal delivery systems. Nanoparticle (NP)-based formulations possess unique characteristics, including small particle size, large surface area, and enhanced stability, which enable improved delivery and sustained release of biological agents [50,51,52,53]. Of these formulations, chitosan-based nanoparticles (CHNPs) have been recognized as carriers for GnRH due to their biocompatibility, biodegradability, and controlled-release properties [54,55]. Moreover, CH-based nanomaterials have exhibited antioxidant, free-radical-scavenging, and anti-inflammatory properties [55]. Previous studies in heat-stressed cattle, including dairy cows and buffaloes, demonstrated that nanofabricated GnRH incorporated within the OVS protocol achieved comparable or improved reproductive responses while reducing the hormone dose by 50% compared with conventional formulations [48,56,57]. However, limited information is available regarding the application of nanofabricated GnRH in dairy heifers, particularly under HS conditions, and the potential impacts of this application on ovarian activity, endocrine status, and metabolic and molecular responses remain unclear.

Considering the adverse effects of HS on ovarian activity and fertility outcomes in dairy heifers, along with the promising benefits of NP-based delivery systems, further studies are recommended to assess innovative reproductive management strategies under HS conditions. We hypothesized that administering a nanofabricated GnRH_a_ formulation at a 50% reduced dose within the OVS protocol could enhance ovarian activity, steroidogenesis, antioxidant status, and related gene expression compared with the standard OVS (SOVS) protocol using the full conventional GnRH_a_ dose. Therefore, the current study aimed to investigate the efficacy of GnRH_a_-loaded chitosan nanoparticles (GnRH_a_–CHNPs) administered at a reduced dose within a modified OVS (MOVS) protocol, compared with conventional GnRH_a_ administration, in Holstein heifers under HS conditions. This study evaluated ovarian activity, hormonal and metabolic status, oxidative stress markers, and circulating expression of folliculogenesis- and apoptosis-related genes that may influence reproductive performance to determine the potential of nanofabricated GnRH_a_ as a novel strategy for improving reproductive efficiency while reducing the hormonal dose in dairy heifers subjected to HS conditions.

## 2. Materials and Methods

### 2.1. Preparation and Characterization of GnRH Nano Formulation

The ionic gelation method was used to fabricate CHNPs, as described by Hassanein et al. [58]. For the preparation of CHNPs as a nanocarrier, pure chitosan (CH; Alpha Chemika, Mumbai, India) with a low molecular weight (300–350 KDa) and a high deacetylation degree of more than 85%, and sodium tripolyphosphate (TPP; Thermo Fisher GmbH, Erlenbachweg 2, Kandel, Germany) were utilized. CH (0.1% *w*/*v*) was dissolved in an aqueous acetic acid solution (1% *v*/*v*) under stirring to produce the polymeric cations. A TPP aqueous solution (0.1 g/dL) was prepared separately. CHNPs were formulated by gradually adding the TPP solution dropwise into the CH solution at a CH-to-TPP ratio of 2:1. GnRH_a_–CHNPs were formed by dropwise addition of a GnRH_a_ analog product (Receptal^®^, containing buserelin acetate, MSD, Intervet International GmbH, Unterschleißheim, Germany) to the prepared CHNPs in a 1:1 ratio under stirring (800 rpm for 60 min) at room temperature. Then, the preparation was incubated overnight to allow hormone adsorption onto the NP surface.

The particle size distribution and specific surface area (SSA) of CHNPs and GnRH_a_–CHNPs were determined using a laser diffraction particle size analyzer (wet mode, Bettersize 2600; Dandong Better size Scientific Ltd., Dandong, China) supplied with an automatic laser centering function. Samples were dispersed in distilled water and subjected to ultrasonic treatment (37 W, 120 s) with stirring at 1500 rpm to prevent aggregation prior to measurement. Several measurements were assessed, including percentile diameters (*D*_10_–*D*_50_–*D*_90_) that describe the cumulative particle size distribution, mean diameter (*D*_1,0_), surface area/Sauter mean diameter (*D*_3,2_), volume/De Brouckere mean diameter (*D*_4,3_), span ((*D*_90_–*D*_10_)/*D*_50_), and SSA.

Hormone loading efficiency (LE%) was measured by centrifuging the NPs from the medium at 1200× *g* for 20 min at 4 °C. The concentration of unconjugated hormone in the supernatant was measured by UV spectrophotometry (Optizen pop, Mecasys Co., Ltd., Daejeon, Republic of Korea) using a standard calibration curve at 280 nm, with the CHNPs supernatant as the blank. LE% was then calculated using the following formula: [total hormone − free hormone in the supernatant/total hormone × 100].

### 2.2. Ethical Approval

The study protocol was approved by the Research Ethics Reviewer Committee of Alexandria University and authorized by the Faculty of Agriculture under reference Alex. Agri. 082512302.

### 2.3. Meteorological Data

**Figure 1:**
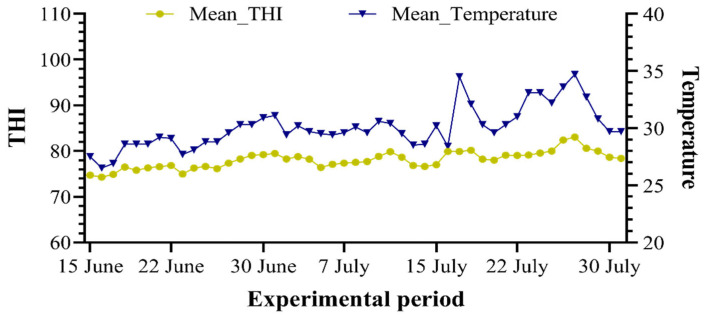
Daily mean ambient temperature (AT) and the temperature–humidity index (THI) were measured over the three weeks prior to the experiment and throughout the experimental period (mid-June to the end of July). A THI value exceeding 72 indicates HS conditions.

The current study was performed under field conditions at Delta Masr Farm for Animal Production in Sadat City, Menoufia Governorate, Egypt (30°21′51.48″ N, 30°44′39.84″ E) during the summer season. Meteorological data, including AT (°C), RH (%), and the THI, were continuously and automatically recorded on-site using a THI-based thermohygrometric control device (New THI System, CMP Impianti S.r.L., Torino, Italy) located at the farm. Data were collected daily for three weeks prior to the experiment and throughout the experimental period, from mid-June to the end of July. Daily averages were calculated from hourly records of AT and the THI (Figure 1).

### 2.4. Animal Management and Experimental Design

All procedures and experimental protocols were performed in accordance with the guidelines outlined in the Guide for the Care and Use of Agricultural Animals in Research and Teaching [59]. The study involved clinically healthy Holstein heifers exposed to HS conditions with an average body weight of 346.4 ± 21.9 kg, aged 17 ± 1.8 months, and a body condition score of 3.0–3.5 on a five-point scale (where 1 = emaciated, 2 = thin, 3 = moderate/optimal, 4 = heavy/fat, and 5 = obese) [60]. Heifers were housed in free-stall, shaded yards and fed a balanced diet formulated to meet their maintenance and nutritional requirements, in accordance with the National Research Council guidelines [61]. Fresh water was provided ad libitum.

**Figure 2:**
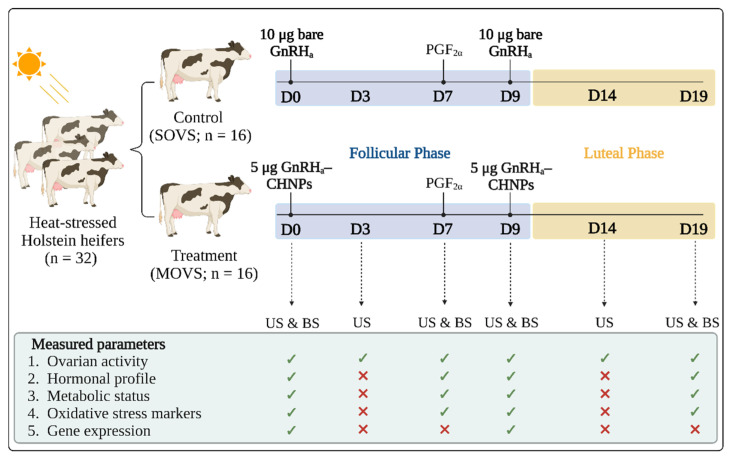
A schematic representation of the experimental design. The control group received the standard Ovsynch protocol (SOVS, n = 16), which included the conventional full dose of bare GnRH_a_ (10 µg/administration). The treatment group received the modified Ovsynch protocol (MOVS; n = 16), which included a 50% reduction in the GnRH_a_–CHNPs dose (5 µg/administration). Additionally, the timing of parameter measurements, including ovarian activity, hormonal profile, metabolic status, oxidative stress markers, and gene expression, across experimental days is also shown. US: Ultrasonography, BS: Blood sampling.

A total of 32 healthy Holstein heifers were randomly allocated into two equal groups (n = 16 each), each subjected to a different Ovsynch (OVS) protocol, as depicted in Figure 2. The first is the control group, which received the SOVS protocol, including two intramuscular (i.m.) injections of 10 μg bare GnRH_a_ (Receptal^®^, Merck, Darmstadt, Germany) on D0 and D9, and a 500 μg i.m. injection of PGF_2α_ (Estrumate^®^, cloprostenol; Vet Pharma GmbH, Friesoythe, Germany) on D7. The second group received a MOVS protocol, in which a 50% reduced dose of GnRH_a_–CHNPs (5 μg) was administered on D0 and D9. In the current study, heifers were not inseminated on D10 because farm management practices restricted AI during periods of severe HS. All cows were subjected to the ovulation synchronization protocol simultaneously, to ensure exposure to comparable environmental conditions throughout the experimental period.

### 2.5. Ultrasound Examination

Ovarian dynamics were monitored using transrectal real-time B-mode veterinary ultrasonography (M12 model, SonoScape, Shenzhen, China) equipped with a 5–7.5 MHz transrectal transducer (Heyi Medical Instrument Co., Ltd., Shanghai, China). Ultrasonographic examinations were performed on D0 (before the first GnRH_a_ administration to check the baseline ovarian status), D3 (to assess the response to the first GnRH_a_ administration), D7 (before the PGF_2α_ administration), D9 (before the second GnRH_a_ administration and to assess the response to the PGF_2α_ administration), D14 (to assess the early luteal phase) and D19 (to assess the mature luteal phase). During each examination, the total number of follicles was recorded and classified based on size into small (≤5 mm), medium (≥5 to <10 mm), and large (≥10 mm) follicles. In addition, the diameter of dominant follicles (DF) on D9, along with the number and diameter of CL, were measured to evaluate ovarian dynamics during the OVS protocols.

### 2.6. Blood Samples Collection

On D0, D3, D7, D9, and D19 of the OVS protocols, two blood samples (5 mL each) were collected from each heifer in the early morning, before feeding, into anticoagulant-containing tubes (EDTA anticoagulant tube). One sample was centrifuged at 800× *g* for 20 min to obtain plasma for subsequent biochemical analyses, while the second sample was stored uncentrifuged for RNA extraction and further gene expression analyses.

### 2.7. Hormonal and Biochemical Analyses

Serum P_4_, E_2_, cortisol, insulin, and IGF-1 concentrations were measured using solid-phase enzyme-linked immunosorbent assay (ELISA) kits (AccuBind^®^ ELISA, Monobind Inc., Lake Forest, CA, USA) according to the manufacturer’s instructions. The lower limits of detection were 0.105 ng/mL, 5.936 pg/mL, 0.054 µg/dL, 0.182 µIU/mL, and 3.80 ng/mL for P_4_, E_2_, cortisol, insulin, and IGF-1, respectively. The intra- and inter-assay coefficients of variation (CV%) were 0.45–5.3% and 0.64–6.4% for P_4_, 5.5–9.9% and 7.5–8.5% for E_2_, 5.7–8.4% and 7.3–8.2% for cortisol, 4.3–6.1% and 3.8–8.2% for insulin, and 2.15–6.78% and 5.86–8.43% for IGF-1, respectively.

Serum glucose concentration was measured calorimetrically using a glucose oxidase-peroxidase (GOD-POD) commercial kit (Reactivos GPL, Barcelona, Spain) according to the manufacturer’s guidelines. The absorbance was measured at 505 nm. The assay’s analytical sensitivity was 1 mg/dL. The intra-assay CV% ranged from 2.03 to 3.09%, whereas the inter-assay CV% ranged from 3.46 to 5.00%.

Additionally, serum oxidative stress biomarkers, including total antioxidant capacity (TAC), superoxide dismutase (SOD), and malondialdehyde (MDA) activity, were determined using colorimetric spectrophotometric methods with commercial kits (Bio-diagnostic^®^, Bio-Diagnostic company, Giza, Egypt) according to the manufacturer’s instructions. TAC was measured at 505 nm, SOD activity at 560 nm, and MDA at 534 nm. Furthermore, the oxidative balance index (OBI) was calculated to estimate the oxidative/antioxidant status as follows: OBI = antioxidant (SOD + TAC)/oxidant (MDA). Higher OBI values indicate stronger antioxidant defense against lipid peroxidation, suggesting improved oxidative balance.

### 2.8. Gene Expression Analysis

#### 2.8.1. RNA Extraction and cDNA Synthesis

Total RNA was isolated from blood samples (n = 3 biological replicates per group) using the RNeasy^®^ Mini Kit (QIAGEN, Hilden, Germany). RNA integrity was verified electrophoretically (RNA Integrity Number, RIN > 8.0) and quantified using a NanoDrop™ 2000 spectrophotometer (Thermo Fisher Scientific, Waltham, MA, USA). First-strand cDNA was synthesized from 500 ng of total RNA using the High-Capacity cDNA Reverse Transcription Kit (Applied Biosystems™, Thermo Fisher Scientific, Waltham, MA, USA) with oligo (dT) primers, according to the manufacturer’s instructions. Reactions (20 µL final volume) were incubated at 25 °C for 10 min, 37 °C for 120 min, and 85 °C for 5 min.

#### 2.8.2. qRT-PCR Analysis

The expression of seven target genes (see Table 1) was quantified using PowerUp™ SYBR™ Green Master Mix (Applied Biosystems™, Thermo Fisher Scientific, Waltham, MA, USA) on a QuantStudio™ 5 Real-Time PCR System. Each 20 µL reaction contained: 10 µL of 2× SYBR Green Master Mix, 0.8 µL of each forward and reverse primer (10 µM), 2 µL of cDNA template (~50 ng/µL), and 6.4 µL of nuclease-free water. The amplification protocol was as follows: initial denaturation at 95 °C for 2 min; 40 cycles of denaturation at 95 °C for 15 s, annealing/extension at 60 °C for 30 s; followed by a melt curve analysis from 60 °C to 95 °C (increment of +0.3 °C/s). *GAPDH* was used as the reference gene for normalization [62], and relative gene expression was calculated using the 2^−ΔΔCT^ method [63]. Three biological replicates and three technical replicates were analyzed for each sample group.

### 2.9. Statistical Analysis

All analyses were performed using IBM SPSS Statistics (Version 24, IBM Corp., SPSS Statistics Inc., Armonk, NY, USA). Normality of continuous variables was assessed using the Shapiro–Wilk test, which confirmed that the data did not follow a normal distribution (*p* < 0.05). Therefore, appropriate data transformations were applied to meet the model’s assumptions of normality and homoscedasticity.

Continuous variables, including follicle and CL diameters and hormone and biochemical profiles, were analyzed using Linear Mixed Models (LMMs) via the MIXED procedure. Treatment (OVS and MOVS), time (D0, D3, D7, D9, D14, and D19 of the protocol), and their interaction were included as fixed factors. Repeated measurements across days were modeled within heifer ID using a first-order autoregressive [AR(1)] covariance structure to account for within-animal correlation over time. Estimated marginal means (EMMs) were evaluated for all fixed factors, and pairwise comparisons were performed using a Bonferroni method.

Count data, such as the numbers of follicles and CL across protocol days, were analyzed using Generalized Linear Mixed Models (GLMMs) with a Poisson distribution and a log link function. Fixed effects included treatment, time, and their interaction. Repeated measures across days were also specified. EMMs were back-transformed to the original scale to facilitate biological interpretation. Bonferroni adjusted-pairwise comparisons were performed after identifying significant effects. Differences were considered statistically significant at *p* ≤ 0.05, and 0.05 < *p* ≤ 0.10 was interpreted as a tendency.

Gene expression differences between experimental groups were analyzed by GraphPad Prism (Version 9.5, GraphPad Software, San Diego, CA, USA) using unpaired, two-tailed Student’s *t*-tests. To account for multiple hypothesis testing across the target genes, *p*-values were adjusted using the Benjamini–Hochberg procedure to control the false discovery rate (FDR), with FDR < 0.05 considered statistically significant. All charts were generated in GraphPad Prism.

## 3. Results

### 3.1. Characteristics of GnRH_a_ Nano Formulation

The physicochemical characteristics of CHNPs before and after GnRH_a_ conjugation to evaluate the effects of GnRH_a_ incorporation on particle size and surface properties are presented in Table 2. Incorporation of GnRH_a_ resulted in a consistent increase in particle size across all metrics. The *D*_10_, *D*_50_, and *D*_90_ values increased from 40, 65, and 171 nm in unconjugated particles to 66, 105, and 273 nm, respectively, following GnRH_a_ incorporation. Similarly, the mean of the particle diameter (*D*_1,0_) enlarged from 94 nm in CHNPs to 151 nm in GnRH_a_–CHNPs. Additionally, noticeable increases were observed in surface- and volume-weighted diameters, with the Sauter mean diameter (*D*_3,2_) increasing from 616 nm to 993 nm and the De Brouckere mean diameter (*D*_4,3_) from 2519 nm to 4188 nm following conjugation.

Despite the increase in particle size, the span value slightly decreased from 1.99 in CHNPs to 1.96 in GnRH_a_–CHNPs. In contrast, the SSA decreased substantially from 3613 to 2241 m^2^/kg following GnRH_a_ conjugation. Moreover, LE% of GnRH_a_ reached a high value of 90.5%, confirming effective conjugation of GnRH_a_ into the CHNPs.

### 3.2. Ovarian Activity

**Figure 3:**
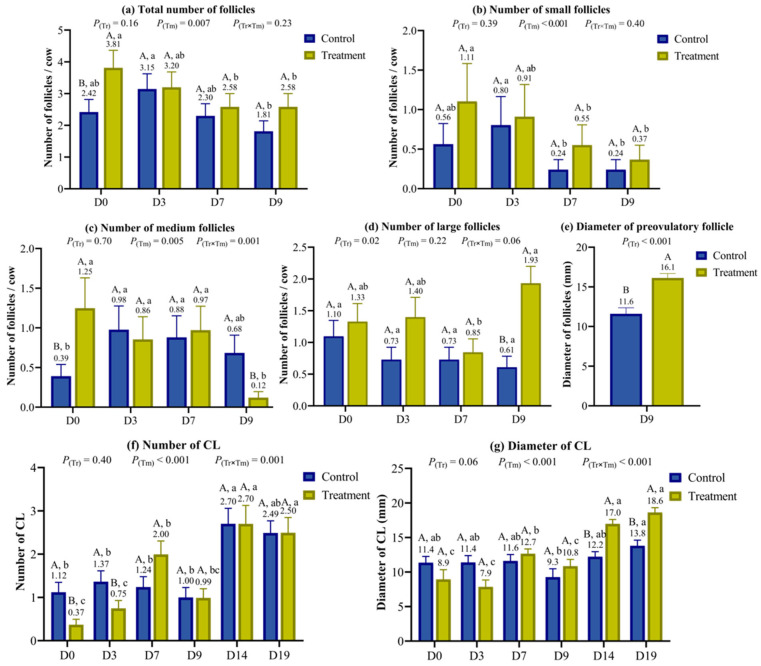
Impact of treatment with the modified OVS (MOVS) protocol containing a reduced dose of GnRH_a_–CHNPs, compared to the standard OVS protocol (SOVS), on ovarian activity across different days of the protocol (D0, D3, D7, D9, D14, and D19). It involves follicular dynamics, including the number of (**a**) total follicles, (**b**) small follicles, (**c**) medium follicles, and (**d**) large follicles, as well as (**e**) the diameter of the dominant follicle (DF). Additionally, luteal dynamics are presented, including (**f**) the number of corpora lutea (CL) and (**g**) CL diameter. Bars marked with different uppercase letters (^A, B^) indicate significant differences between treatments on the same day. In contrast, bars marked with different lowercase letters (^a, b, c^) indicate significant differences among days within the same treatment.

The changes in ovarian dynamics, including follicular and luteal development, across protocol days between experimental groups are illustrated in Figure 3. The total number of follicles, as shown in Figure 3a, was significantly affected by time (*p* = 0.007). However, neither the treatment nor the time × treatment interaction was significant (*p* > 0.05). Although the MOVS group showed a significantly higher follicle count than the SOVS group at D0 (3.81 ± 0.55 vs. 2.42 ± 0.40, respectively; *p* < 0.05), follicular numbers became comparable (*p* > 0.05) between groups across the subsequent protocol days (D3, D7, and D9) after the protocol initiation.

Similarly, the number of small follicles was significantly affected by time (*p* < 0.001) but not by treatment or treatment × time interaction (*p* > 0.05; Figure 3b). Before the initiation of the protocols (D0), MOVS-treated heifers had a non-significantly higher count (1.11 ± 0.48) than SOVS-treated heifers (0.56 ± 0.26). Conversely, during the subsequent days of the protocol (D3, D7, and D9), small follicle counts were similar in both groups and declined progressively over time.

Regarding the number of medium follicles (Figure 3c), treatment did not significantly affect the counts (*p* = 0.70); however, both time and interaction were significant (*p* = 0.005 and *p* = 0.001, respectively). At D0, the MOVS group significantly (*p* < 0.05) outperformed the SOVS group (1.25 ± 0.38 vs. 0.39 ± 0.15). By D3 and D7, both groups showed comparable values that were not significantly different. In contrast, by D9, the MOVS group showed fewer medium follicles (0.12 ± 0.08) than the control group (0.68 ± 0.22; *p* < 0.05).

The number of large follicles was significantly affected by the treatment (*p* = 0.02), whereas time (*p* = 0.22) and the interaction (*p* = 0.06) did not exhibit significant effects (Figure 3d). While both groups exhibited comparable counts in the early days of the protocol, including D0, D3, and D7, the MOVS group showed a significantly higher value (1.93 ± 0.27) than the SOVS group (0.61 ± 0.17) by D9 (*p* < 0.05). Additionally, the diameter of the DF on D9, as presented in Figure 3e, was significantly greater (*p* < 0.001) in the MOVS group (16.13 ± 0.57 mm) than in the SOVS group (11.61 ± 0.75 mm).

The luteal activity, including CL number and diameter, is illustrated in Figure 3f and Figure 3g, respectively. The number of CLs was not affected by treatment (*p* = 0.40) but was significantly affected by time (*p* < 0.001) and showed a significant interaction (*p* = 0.001). At D0, SOVS-treated heifers had a higher number of CL than MOVS-treated heifers (1.12 ± 0.23 vs. 0.37 ± 0.13, respectively, *p* < 0.05). This pattern also remained at D3. However, by D7, the MOVS group significantly exceeded the SOVS group (2.00 ± 0.31 vs. 1.24 ± 0.24, respectively). Following PGF_2α_ administration, both groups displayed a marked decline by D9, with no significant difference between the groups. At D14 and D19, following ovulation, both groups showed values nearly double those at D9, with no significant differences between groups at these later time points.

On the other hand, the diameter of CL tended to be affected by treatment (*p* = 0.06) but was significantly affected by time and by the treatment × time interaction (both *p* < 0.001). At D0, the SOVS group recorded a numerically larger CL diameter (11.35 ± 0.92 mm) than the MOVS group (8.94 ± 1.40 mm). A similar trend also continued at D3. By D7, the treatment group showed a slight increase in CL diameter compared to the SOVS group (12.65 ± 0.70 vs. 11.62 ± 0.92, respectively). At D9, both groups showed a significant decline in response to PGF_2α_ administration. However, a slight numerical increase was observed in the MOVS group (9.27 ± 1.21 mm in the SOVS vs. 10.85 ± 0.99 mm in the MOVS). As the protocol progressed, by D14, MOVS-treated heifers had a markedly (*p* < 0.05) larger CL diameter (16.97 ± 0.65 mm) than controls (12.22 ± 0.81 mm). This trend continued through D19, with the MOVS group remaining substantially higher than the SOVS group (18.63 ± 0.70 mm vs. 13.81 ± 0.81 mm; *p* < 0.05).

### 3.3. Reproductive and Metabolic Hormone Profiles

**Figure 4:**
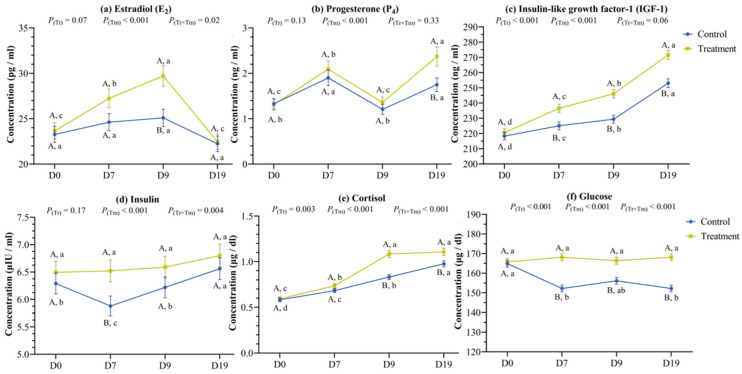
Impact of treatment with modified OVS (MOVS) protocol containing a reduced dose of GnRH_a_–CHNPs, compared to the standard OVS protocol (SOVS) on reproductive and metabolic hormones profile across different days of the protocol (D0, D7, D9, and D19), including (**a**) estradiol (E_2_), (**b**) progesterone (P_4_), (**c**) insulin like growth factor-1, (**d**) insulin, (**e**) cortisol, and (**f**) glucose. Different uppercase letters (^A, B^) indicate significant differences between treatments on the same day, while different lowercase letters (^a, b, c, d^) indicate significant differences among days within the same treatment (*p* < 0.05).

The effects of treatment, time, and their interaction on reproductive and metabolic hormones are shown in Figure 4. For ovarian hormones, E_2_ tended to be affected by treatment (*p* = 0.07) but was significantly affected by time (*p* < 0.001) and by the interaction between treatment and time (*p* = 0.02). Both groups were initiated with similar baseline values at D0. By D7, E_2_ concentrations increased in both groups, with slightly higher levels in the MOVS group (27.24 ± 1.04 pg/mL) than in the SOVS group (24.62 ± 0.94 pg/mL). However, the difference between groups was not significant (*p* > 0.05). This difference became more pronounced by D9 (*p* < 0.05), with the MOVS group peaking at 29.72 ± 1.13 pg/mL, while the SOVS group showed only a modest increase to 25.10 ± 0.95 pg/mL. In contrast, E_2_ concentrations declined substantially in both groups by D19 (~22.0 pg/mL in each), with no significant difference between them.

Additionally, P_4_ concentrations were not significantly affected by treatment (*p* = 0.13) but were strongly affected by time (*p* < 0.001), with no significant interaction (*p* = 0.33). At D0, both groups had comparable P_4_ values. P_4_ increased significantly in both groups, with a greater increase in the MOVS group than in the SOVS group (2.09 ± 0.18 vs. 1.90 ± 0.17 ng/mL, respectively). At D9, both groups exhibited a significant decline in response to PGF_2α_ administration (1.21 ± 0.11 in the control and 1.36 ± 0.12 ng/mL in the treatment group), with no difference between groups. By D19, both groups showed a significant increase following the second GnRH_a_ administration, with the MOVS-treated heifers showing a significantly higher level (2.37 ± 0.21 ng/mL) than the SOVS-treated heifers (1.75 ± 0.15 ng/mL).

IGF-1 concentrations were strongly affected by both treatment (*p* < 0.001) and time (*p* < 0.001), with a tendency in their interaction effect (*p* = 0.06). Baseline levels were comparable between groups at D0. As protocol progressed, IGF-1 concentrations increased in both groups, with a significantly greater rise in the MOVS group (236.6 ± 2.6 ng/mL) than in the SOVS (225.1 ± 2.5 ng/mL). This difference was significantly (*p* < 0.05) broadened by D9 (246.61 ± 2.7 ng/mL in the MOVS group vs. 229.4 ± 2.6 ng/mL in the SOVS group) and became most pronounced at D19, where IGF-1 reached its peak and remained significantly higher (*p* < 0.05) in the MOVS group compared to the SOVS group (271.6 ± 2.9 vs. 253.1 ± 2.8 ng/mL, respectively).

Moreover, insulin concentrations were not significantly affected by treatment (*p* = 0.17) but showed a significant time effect (*p* < 0.001) and a significant interaction between time and treatment (*p* = 0.004). At D0, insulin levels did not differ between groups. By D7, insulin remained stable in the MOVS group (5.88 ± 0.18 µIU/mL), while a significant reduction (*p* < 0.05) was observed in the SOVS group (6.52 ± 0.20 µIU/mL). In contrast, at D9, the SOVS group showed a significant increase (6.22 ± 0.19 µIU/mL, *p* < 0.05). In contrast, the MOVS group maintained a higher value of 6.59 ± 0.20 µIU/mL. By D19, insulin levels increased in both groups, reaching 6.56 ± 0.20 µIU/mL in the SOVS group and 6.80 ± 0.21 µIU/mL in the MOVS group, with no significant difference between the groups.

For cortisol, all fixed effects, including treatment (*p* = 0.003), time (*p* < 0.001), and their interaction (*p* < 0.001), were significant. Similar values were measured in both groups at D0. By D7, cortisol concentrations increased gradually in both groups, with no significant between-group differences, reaching 0.68 ± 0.02 µg/dL in the SOVS group and 0.74 ± 0.03 µg/dL in the MOVS group. By D9, although levels continued to rise in both groups, the MOVS group exhibited significantly higher (*p* < 0.05) levels than the SOVS group (1.08 ± 0.04 vs. 0.83 ± 0.03 µg/dL, respectively). By D19, cortisol concentrations continued to increase in the SOVS group (0.98 ± 0.03 µg/dL), whereas the MOVS group showed only a slight increase (1.11 ± 0.04 µg/dL); nevertheless, cortisol concentrations remained significantly higher in the MOVS group (*p* < 0.05).

Similarly, glucose concentrations were significantly affected by treatment, time, and their interaction (*p* < 0.001). Baseline levels were similar between groups (~165.0 µg/dL). At D7, glucose decreased in the SOVS group to 152.2 ± 1.7 µg/dL, whereas it remained unchanged in the MOVS group at 168.1 ± 1.8 µg/dL, resulting in significantly higher levels in the SOVS group (*p* < 0.05). This difference persisted across D9 and D19, with the treatment group consistently maintaining higher glucose concentrations than the control group.

### 3.4. Oxidative Stress and Antioxidant Indices

**Figure 5:**
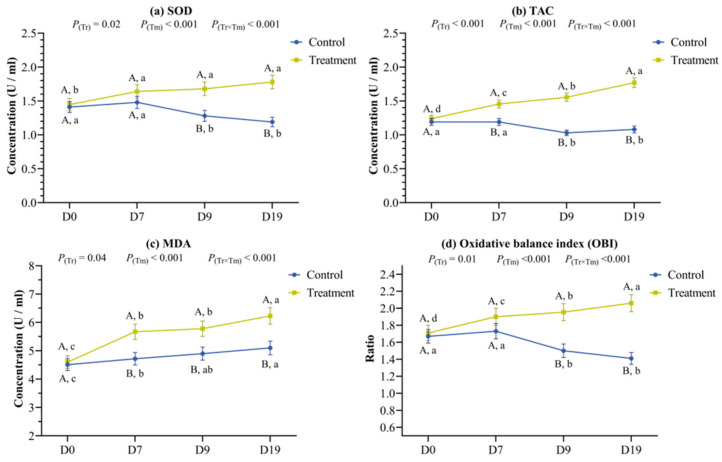
Impact of treatment with the modified OVS (MOVS) protocol containing a reduced dose of GnRH_a_–CHNPs, compared to the standard OVS (SOVS) protocol, on antioxidant and oxidative stress markers across different days of the protocol (D0, D7, D9, and D19), including (**a**) superoxide dismutase (SOD), (**b**) total antioxidant capacity (TAC), (**c**) malondialdehyde (MDA) and (**d**) oxidative balance index (OBI). Different uppercase letters (^A, B^) indicate significant differences between treatments on the same day, while different lowercase letters (^a, b, c, d^) indicate significant differences among days within the same treatment (*p* < 0.05).

Figure 5 depicts the impact of fixed effects, including treatment, time, and their interaction, on oxidative stress and antioxidant indices, including SOD, TAC, MDA, and OBI, all of which were significantly influenced (*p* < 0.05).

SOD activity was similar between groups at D0. By D7, the treatment group showed significantly higher (*p* < 0.05) SOD activity than the SOVS group (1.64 ± 0.10 vs. 1.48 ± 0.09 U/mL, respectively). This difference persisted through D9 and D19 (*p* < 0.05), as SOD activity gradually declined in the SOVS heifers, reaching 1.19 ± 0.07 U/mL, while continuing to increase slightly in the MOVS group, peaking at 1.78 ± 0.10 U/mL.

A similar pattern was observed for TAC. Both groups exhibited comparable TAC values at D0. From D7 onward, TAC increased steadily in the MOVS group, reaching its maximum level of 1.77 ± 0.07 U/mL at D19. Conversely, the SOVS group remained relatively stable by D7 (1.19 ± 0.05 U/mL). Subsequently, they declined slightly at D9 and D19, reaching their lowest value of 1.08 ± 0.05 U/mL, resulting in significantly higher TAC levels (*p* < 0.05) in the MOVS group at all time points following the initiation day.

MDA levels were similar before protocol initiation. By D7, both groups showed a gradual increase, with significantly higher MDA levels (*p* < 0.05) in the MOVS group (5.67 ± 0.27 U/mL) than in the SOVS group (4.72 ± 0.22 U/mL). This difference persisted at D9 and became more pronounced by D19 (5.10 ± 0.24 U/mL in the SOVS vs. 6.23 ± 0.29 U/mL in the MOVS group), indicating greater lipid peroxidation in the MOVS group.

Consequently, OBI values were similar between groups on D0 and D7. By D9 and D19, OBI increased continuously in the MOVS group, reaching its peak of 2.06 ± 0.10 at D19, while the SOVS group gradually declined to its lowest value of 1.41 ± 0.07. This resulted in significantly higher OBI values (*p* < 0.05) in the MOVS group at these points.

### 3.5. Gene Expression

**Figure 6:**
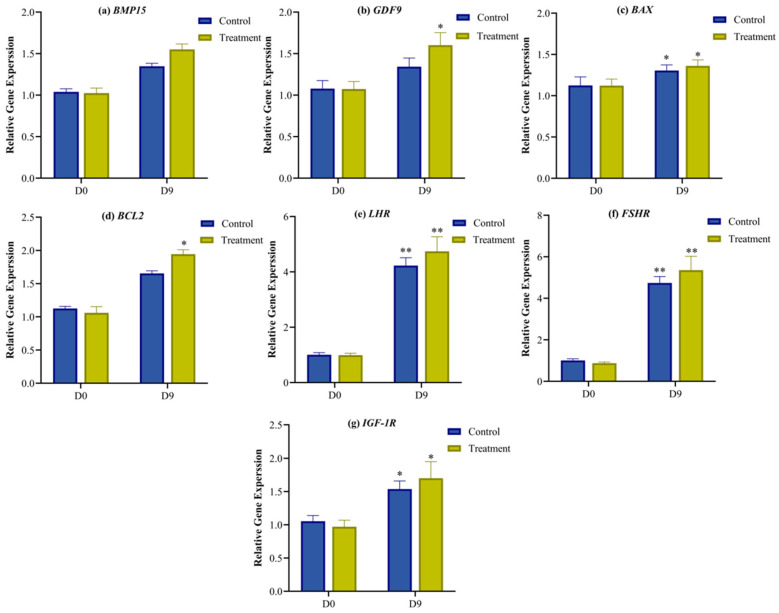
Impact of treatment with the modified OVS (MOVS) protocol containing a reduced dose of GnRH_a_–CHNPs, compared to the standard OVS (SOVS) protocol, on relative transcript expression of seven genes in peripheral blood on D0 and D9. Data normalized to *GAPDH* (2^−ΔΔCT^), mean ± SD (n = 3). * *p* < 0.05, ** *p* < 0.01 indicate significant differences between days within each group for each gene; (**a**) *BMP15*, (**b**) *GDF9*, (**c**) *BAX*, (**d**) *BCL2*, (**e**) *LHR*, (**f**) *FSHR,* and (**g**) *IGF-1R*.

Baseline transcript levels (D0) of all seven target genes were similar between the SOVS and MOVS groups, ranging from 0.87 to 1.35, with no significant differences detected (*p* > 0.05 for all genes; Figure 6). At D9, both groups showed increased expression of several genes in circulation. In the SOVS group, *LHR* (Figure 6e) and *FSHR* (Figure 6f) were highly upregulated (4.2-fold, *p* < 0.001, and 4.7-fold, *p* < 0.001, respectively). Moderate increases (1.25- to 1.47-fold, *p* < 0.05) were also observed for *BMP15* (Figure 6a), *GDF9* (Figure 6b), *BCL2* (Figure 6d), and *IGF-1R* (Figure 6g).

In the MOVS group, circulating transcript levels of *LHR* increased from 0.96 to 4.74 (4.9-fold, *p* < 0.001) and *FSHR* from 0.87 to 5.36 (6.2-fold, *p* < 0.001) between D0 and D9. However, when comparing MOVS-treated heifers directly to SOVS-treated heifers at D9, the fold changes were modest (1.04–1.19), and none reached statistical significance (*p* > 0.05). For example, treatment increased circulating transcriptional patterns of *BMP15* to 1.55 (1.15-fold), *GDF9* to 1.60 (1.19-fold), *LHR* to 4.74 (1.12-fold), and *FSHR* to 5.36 (1.13-fold) relative to control. *BAX* expression (Figure 6c) was unchanged between groups.

These findings confirm that both protocols effectively stimulate the circulating transcription of gonadotropin receptor genes. Although the MOVS protocol did not significantly alter circulating transcript levels compared to the SOVS protocol, it maintained comparable gene expression profiles at a 50% reduced GnRH_a_ dose, supporting its feasibility under heat-stress conditions.

## 4. Discussion

The current study investigated the effect of a 50% reduced dose of GnRH_a_–CHNPs within the OVS protocol on ovarian activity, hormonal and metabolic status, oxidative stress markers, and folliculogenesis- and apoptosis-related gene expression in dairy heifers exposed to HS conditions.

CHNPs have emerged as promising carriers for various bioactive molecules due to their ability to protect encapsulated molecules from enzymatic degradation, enhance bioavailability, provide sustained release, facilitate transport across biological barriers, and improve cellular uptake [52,55]. Physical features of NPs significantly affect their functional characteristics, particularly particle size. This feature is a key factor in determining the ability to penetrate biological barriers and achieve intracellular uptake [55].

In the present study, the physicochemical characterization of CHNPs and GnRH_a_–CHNPs revealed noticeable changes in particle size distribution and surface properties following conjugation of GnRH_a_ into the CHNPs matrix. The percentile diameters, including *D*_10_, *D*_50_, and *D*_90_, as well as *D*_1,0_, increased markedly after GnRH loading. These results indicate that the GnRH_a_ formulation was successfully conjugated within or absorbed onto the CHNPs matrix, resulting in enlargement of the NPs, possibly due to swelling of the polymeric matrix or surface-coating effects. This finding aligns with previous studies showing an increase in particle size after GnRH_a_ loading into CHNPs compared with unconjugated CHNPs [57,58,69,70].

Although particle size increased after conjugation, the span values remained nearly stable, with only a slight decrease from 1.99 to 1.96 before and after conjugation, respectively. This finding suggests that GnRH_a_ conjugation did not markedly broaden particle size distribution or induce excessive aggregation, indicating acceptable formulation homogeneity and good physical stability during the loading process.

Conversely, the SSA decreased by ~38% following GnRH_a_ loading. This reduction may be associated with an increase in particle size and a subsequent decrease in the exposed surface area per unit mass. Additionally, GnRH_a_ molecules may fill surface pores or interact with active binding sites on the CHNPs matrix, thereby reducing the accessible surface area through surface-modification effects [71].

Notably, the mean particle size after GnRH_a_ loading remained within the nanoscale range, considered beneficial for biological applications and cellular uptake. Nanoparticle size is a critical determinant for crossing biological barriers and achieving intracellular uptake [55,72]. Most NPs used for drug delivery range from 50 to 250 nm, facilitating effective transport across biological barriers and improving cellular uptake [73,74]. Based on the literature, the GnRH_a_–CHNPs formulation with a size of ~150 nm remains appropriate for drug delivery applications.

The hormone LE% of the GnRH_a_–CHNPs reached 90.5%, indicating successful incorporation of GnRH_a_ with CHNPs and emphasizing the high loading capacity of CH as a nanocarrier. This observation aligns with previous studies reporting high LE values for various GnRH_a_ formulations incorporated into CHNPs, typically ranging from 87.7% to 91.2% [56,57,58,69,70,75,76]. Such a high LE% indicates that the largest proportion of GnRH_a_ used during preparation was effectively incorporated into or adsorbed onto the CHNPs matrix. Furthermore, the interaction between the hormone and nanocarriers may enhance controlled release behavior, increase bioavailability, improve targeting efficiency, and potentially reduce the required dose [77]. This result may also explain the observed reduction in SSA, as high hormone LE% could cover the NP surfaces and fill internal polymeric pores.

Overall, the present physicochemical observations suggest successful NP formulation and effective hormone conjugation, resulting in a physically stable and effective delivery system. These characteristics support the potential application of GnRH_a_–CHNPs as a controlled-release delivery system to improve reproductive management in dairy heifers subjected to HS conditions.

Integrating GnRH_a_–CHNPs within the OVS protocol markedly affected the ovarian activity in heifers. Before protocol initiation (D0), both groups exhibited generally comparable ovarian activity, although the control group had a significantly higher total follicle count than the treatment group. This difference is likely due to the higher number of medium-sized follicles measured at D0. However, following protocol initiation, these differences gradually disappeared. During the early phase of the OVS protocol (up to D7), both groups exhibited comparable follicular recruitment and follicular wave emergence, as evidenced by similar numbers of small, medium, and total follicles on D3 and D7. These findings suggest that the sustained release of the reduced GnRH_a_–CHNPs dose-maintained gonadotropin stimulation and supported follicular responsiveness during the recruitment phase, similarly to the full dose of conventional GnRH_a_.

By D9, the MOVS-treated heifers showed a lower count of medium follicles and a higher count of large follicles, along with significantly larger DF diameters than the SOVS group. The reduction in medium follicle count, accompanied by increased large follicles, suggests more efficient transition of recruited follicles toward the dominance/preovulatory stage in the MOVS-treated heifers. These observations suggest that the MOVS enhanced follicular growth, dominance, and ovulatory readiness by improving synchronization efficiency compared with the SOVS protocol.

HS is known to impair DF development and follicular steroidogenesis during summer conditions [49,78]. Therefore, the enhanced DF growth observed in the MOVS-treated heifers may indicate improved follicular competence and ovarian responsiveness despite HS conditions. The hormonal pattern further supported this interpretation, as treated heifers exhibited increased E_2_ concentrations during follicular development and dominance. Because E_2_ synthesis is directly associated with follicular growth and gonadotropin responsiveness, high E_2_ concentrations generally reflect improved follicular competence and maturation [79].

These observed beneficial effects may be attributed to the GnRHa–CHNPs, which likely protected the hormone from enzymatic degradation and enabled prolonged release. This sustained release consequently triggers the HPO axis and extends LH secretion [55,58]. Contiguous LH availability is essential for the final maturation of follicles and development of a competent ovulatory follicle [80,81]. The size of follicles at the time of AI is a critical determinant of E_2_ concentrations, ovulation quality, and the hormonal environment essential for successful fertilization and PR establishment [82]. Larger DF generally results in higher E_2_ levels, enhancing estrous expression, LH surge responsiveness, and oocyte competence, thereby improving fertility outcomes [82]. Therefore, heifers with well-developed ovulatory follicles are more likely to achieve higher fertility rates in TAI synchronization protocols.

These observations are consistent with previous reports documented in other livestock species. In rabbits, nanofabricated GnRH_a_ successfully induced ovulation at lower dosages while triggering earlier LH surges compared with conventional GnRH_a_ administration [58]. Similarly, incorporation of nanofabricated GnRH_a_ within OVS protocols enhanced follicular development in anestrous buffalo cows and heat-stressed dairy cows, leading to increased DF size and improved fertility outcomes [48,56]. In goats, nanofabricated GnRH_a_ increased both follicle numbers and follicle diameter, along with high levels of E_2_ and nitric oxide (NO), which are associated with improved ovarian blood flow and steroidogenesis due to NO’s vasodilatory properties [83,84]. Overall, these reports support the hypothesis that nanofabricated GnRH_a_ enhances ovarian responsiveness through improved hormone stability and bioavailability.

Luteal dynamics further supported the beneficial effects of MOVS. In our study, both groups demonstrated effective luteolysis following PGF_2α_ administration, as evidenced by regression in the number and diameter of CL on D9 compared with D7 [85]. However, the MOVS-treated heifers showed a numerically larger diameter of CL during the follicular phase, along with numerically higher P_4_ concentrations. High P_4_ concentrations during the follicular phase are known for improving follicular turnover and facilitating the emergence of a new follicular wave [86,87]. These effects may also be associated with increased DF diameter and high levels of E_2_ and NO, which collectively contribute to improved ovarian blood flow [48,56,83].

Following ovulation, the MOVS-treated heifers exhibited significantly larger CL during the early and mid-luteal phases (D14 and D19), compared to the control heifers. This finding is also supported by high P_4_ concentrations, indicating enhanced luteal development and function. Administration of GnRH around the time of ovulation stimulates LH release and promotes luteinization of follicular cells [51]. Therefore, sustained release of GnRH_a_ from GnRH_a_–CHNPs likely prolonged LH secretion and enhanced follicular luteinization, resulting in larger and more functional CL [76,83]. Notably, because CL diameter is directly correlated with the size of DF and the content of GCs [48,88,89], larger CLs are associated with increased levels of P_4_, promoted uterine receptivity, and improved embryo survival [90]. Furthermore, high P_4_ levels during the mid- and late-luteal phases are essential for regulating endometrial gene expression, uterine secretions, and interferon-tau signaling, all of which are fundamental for maintaining PR in cattle [91,92]. Comparable results have been observed in heat-stressed dairy cattle, buffaloes, and goats, where the use of nanofabricated GnRH_a_ effectively enhanced CL development, P_4_ concentrations, and subsequent fertility outcomes despite reduced hormone dosage [48,56,57,83].

Moreover, metabolic indicators supported the increased ovarian activity observed in MOVS-treated heifers. Increased concentrations of IGF-1, insulin, and glucose suggest improved metabolic support for follicular development, steroidogenesis, and ovarian activity. The synchronized increase in metabolic markers suggests that GnRH_a_–CHNPs promoted a more favorable endocrine-metabolic environment that supported follicular growth, ovulation, and subsequent luteal development. This improved metabolic status may partially explain the enhanced follicular growth, DF diameter, and high E_2_ levels observed during the follicular phase.

Among these metabolic factors, IGF-1 appears to play a critical role in regulating ovarian function. Previous studies reported that IGF-1 potentiates gonadotropin action by stimulating GC proliferation and steroidogenesis, and by enhancing E_2_ synthesis. It also acts synergistically with follicle-stimulating hormone (FSH) and LH to support follicular differentiation, oocyte competence, and luteal function [93,94]. Additionally, IGF-1 enhances aromatase activity, inhibin secretion, and LH receptor development within follicular cells [95]. Moreover, IGF-1 exerts protective effects by reducing DNA fragmentation in heat-stressed oocytes, thereby supporting oocyte quality under HS conditions [96]. Additionally, IGFs contribute to luteal development through boosting angiogenesis, luteinization, and P_4_ production during early CL formation [97,98].

Similarly, improved insulin and glucose availability may also support the increased energy demands associated with accelerated follicular growth, ovulation, and CL formation. Insulin plays a key role in glucose uptake, energy metabolism, and thermogenic regulation, and adequate insulin levels are considered important for minimizing heat-induced cellular damage [99,100]. Previous studies reported that acute HS changes insulin dynamics and glucose metabolism as adaptive responses aimed at maintaining energy homeostasis under challenging environmental conditions [100,101,102,103].

Interestingly, cortisol concentrations were also increased in MOVS-treated heifers. Cortisol is a major glucocorticoid involved in HPA axis activation and in maintaining metabolic homeostasis during HS [104]. Previous studies reported that increased cortisol levels stimulate glycogenolysis and improve blood glucose levels, enabling animals to better cope with HS conditions [104,105,106,107]. In the present study, a marked increase in cortisol was accompanied by elevated glucose and IGF-1 levels, enhanced follicular growth, increased E2 synthesis, and improved luteal development, suggesting that cortisol remained within the physiological adaptive range rather than reflecting stress-induced damage. Acute HS has been associated with transient elevations in cortisol required to maintain metabolic stability and thermoregulation, whereas prolonged HS may suppress cortisol secretion and impair reproductive performance [101,104,108]. Therefore, the high cortisol concentration observed in MOVS-treated heifers may reflect enhanced adaptive metabolic activation rather than detrimental stress.

Oxidative stress and antioxidant responses are critical regulators of follicular integrity and oocyte quality during summer conditions. As the protocol progressed, MOVS-treated heifers showed a gradual increase in SOD activity, TAC, and OBI compared with the control group, indicating enhanced antioxidant defense mechanisms during both the follicular and luteal phases. Follicular growth, ovulation, and luteal development are physiologically associated with ROS generation; however, excessive ROS accumulation during HS impairs oocyte maturation, follicular dynamics, and steroidogenesis [26,29,31,109,110,111]. Therefore, adequate antioxidant protection is necessary to maintain cellular homeostasis and normal ovarian activity. Our findings are consistent with previous reports demonstrating that antioxidant enzymes such as SOD and CAT play protective roles against HS-induced oxidative damage by regulating oxidative pathways and suppressing ROS accumulation in GCs [112,113].

Although MDA concentrations were higher in MOVS-treated heifers during the experimental period, this rise may reflect not only potential oxidative damage but also increased metabolic and steroidogenic activity associated with enhanced ovarian activity. Notably, the improved antioxidant profile observed in MOVS-treated heifers may also be related to the fundamental biological properties of CHNPs. Recent studies have demonstrated that CH-based nanomaterials possess antioxidant, free radical-scavenging, and anti-inflammatory properties that reduce oxidative injury and enhance cellular resistance under HS conditions [114,115]. CHNPs have also been reported to enhance antioxidant enzyme activity and modulate oxidative stress-related pathways in biological tissues [55]. Therefore, the beneficial effects observed in the MOVS-treated group may result from the combined effects of sustained GnRH_a_ release and the antioxidant-supportive properties of CHNPs, which may all help preserve follicular function and improve ovarian resilience during the HS.

Because oxidative stress is a major trigger for apoptosis under HS conditions, the improved antioxidant profile observed in MOVS-treated heifers may partly explain the modulation of apoptotic pathways detected in the present study. Both groups showed significantly higher *BAX* expression on D9 than on D0; however, *BCL2* expression increased significantly only in the treated group. This pattern indicates maintenance of follicular survival signaling rather than excessive apoptotic activation. Previous reports have shown that ROS accumulation and oxidative stress activate apoptotic pathways by altering the balance of regulated apoptotic genes [26,31,32,34,113]. HS has also been shown to upregulate *BAX*, caspases, and other apoptosis-related factors in bovine GCs and oocytes [31,116,117]. Therefore, the ability of MOVS-treated heifers to maintain stable apoptotic signaling, along with improved antioxidant status and ovarian dynamics, may reflect a protective ovarian environment during summer conditions.

At the molecular level, the MOVS protocol was associated with a numerical increase in the circulating expression of *BMP15*, *GDF9*, *FSHR*, *LHR*, and *IGF-1R*. However, these differences were not statistically significant compared with the control group. Nevertheless, the consistent upregulation of these genes’ peripheral blood transcripts, together with the observed improvements in ovarian activity, hormonal profile, and oxidative status, suggests that these molecular changes may be biologically meaningful under an HS environment.

Among the detected genes, *BMP15* and *GDF9* are critical oocyte-derived growth factors involved in GC proliferation, follicular maturation, and ovulatory competence [118]. *GDF9* promotes GC proliferation and differentiation during early follicular development and supports communication between the oocyte and surrounding somatic cells, which is crucial for oocyte maturation and developmental competence [119]. Similarly, *BMP15* acts synergistically with *GDF9* to regulate folliculogenesis, GC function, and oocyte maturation [120]. Previous reports have demonstrated that HS suppresses *BMP15* and *GDF9* expression, impairs oocyte competence, and alters follicular survival pathways [32,36]. Therefore, the tendency toward increased circulating expression of these folliculogenesis-related genes in MOVS-treated heifers could help explain the enhanced follicular activity, larger DF diameter, and increased E_2_ synthesis observed in our study.

Similarly, the increased circulating expression of *FSHR* and *LHR* suggests enhanced gonadotropin responsiveness in MOVS-treated heifers. Both receptors are necessary regulators of ovarian activity [38]. *FSHR* mediates the action of FSH in GCs, promoting follicular growth, aromatase activity, and E_2_ production [31], whereas *LHR* is important for LH-mediated follicular maturation, ovulation, luteinization, and CL development [26,121]. HS has been shown to alter the expression of both receptors, resulting in a disruption of follicular dynamics and steroidogenesis [37,39]. Consequently, the tendency toward increased circulating *FSHR* and *LHR* expression in MOVS-treated heifers may support improved ovarian responsiveness and enhanced ovulatory and luteal responses during the protocol.

Both groups exhibited upregulated *IGF-1R* expression in peripheral blood on D9 compared to D0, with a numerical increase observed in MOVS-treated heifers. The tendency toward upregulation of *IGF-1R* further supports enhanced metabolic and endocrine signaling within the follicular phase. IGF-1 acts synergistically with gonadotropins to regulate GC and TC proliferation, survival, and steroidogenesis [122]. Previous studies have reported that the lack or loss of *IGF-1R* expression in GC results in infertility, impaired antral follicle development, and increased follicular apoptosis, highlighting the vital role of IGFs in ovarian function [122,123,124]. Collectively, these circulating molecular responses support the physiological, endocrine, metabolic, and antioxidant improvements observed throughout the MOVS protocol under challenging environmental conditions. 

## 5. Conclusions

The current study demonstrated that a 50% reduction in GnRH_a_–CHNPs dosage within the MOVS protocol enhanced ovarian responses in heifers under an HS environment compared with the SOVS protocol. Nanofabricated GnRH_a_ treatment improved follicular growth, DF development, CL characteristics, endocrine responses, metabolic status, and oxidative status. These improvements were also associated with numerical changes in circulating expression patterns of folliculogenesis-related genes, including *BMP15*, *GDF9*, *FSHR*, *LHR*, and *IGF-1R*, as well as in apoptosis-related genes, with increased *BCL2* expression while maintaining stable *BAX* expression, suggesting a potential supportive molecular response under HS conditions. The enhanced ovarian responses observed in the MOVS-treated group may be attributed to sustained GnRH_a_ release, increased hormone bioavailability, and the possible antioxidant properties of CHNPs. The MOVS protocol partially alleviates endocrine, metabolic, and oxidative disturbances associated with summer HS, thereby improving ovarian activity compared with the SOVS protocol. Although molecular markers did not reach statistical significance, the consistent directional trends, along with physiological improvements, support the biological relevance of nanofabricated GnRH_a_ administration. Therefore, the observations of the present study support the potential application of nanotechnology-based hormone delivery systems as an advanced reproductive management approach under challenging environmental conditions.

Further studies are recommended to evaluate different doses of nanofabricated GnRH_a_ within the OVS synchronization protocol. We suggest that increasing the administered dose of nanofabricated GnRH_a_ with CHNPs may enhance the magnitude of endocrine, ovarian, and antioxidant responses, particularly the molecular responses, and could potentially maximize reproductive performance under HS conditions. Additionally, studies involving larger populations, evaluations of ovulation timing, LH patterns, fertility outcomes, and long-term reproductive performance are required to optimize the practical application of nanofabricated GnRH_a_-based synchronization systems in livestock production.

## Figures and Tables

**Table 1:** Investigated key genes and the reference gene involved in the current study.

No.	Gene	GenBank Accession	Primer Sequence (5′→3′)	Product Size	Ref.
1	*BMP15*	AY572412	ATCATGCCATCATCCAGAACC	72 bp	[64]
			TAAGGGACACAGGAAGGCTGA		
2	*GDF9*	AB058416	AGCGCCCTCACTGCTTCTATAT	80 bp	
			TTCCTTTTAGGGTGGAGGGAA		
3	*BAX*	NM_173894.1	CCCGAGTTGATCAGGACCAT	96 bp	[65]
			CACTCCAGCCACAAAGATGG		
4	*BCL2*	NM_001166486.1	CTTTGTGGAGCTGTATGGC CCAGATAGGCACCCAGGG	133 bp	
5	*LHR*	U20504.2	TTCTGCTTACCCAAGACACTC	106 bp	[66]
			TAATCAGCCAAATCAGGACTC		
6	*FSHR*	NM_174061	CATGCTCATCTTCACCGACTT	112 bp	[67]
			GACCAGGAGGATCTTTGACTTG		
7	*IGF-1R*	NM_001244612.1	GTATGGAGGAGCCAAGCTAAA	123 bp	[68]
			GTCTTGGCCTGAACGTAGAA		
Ref.	*GAPDH*	NM_001034034.3	GCCATCAATGACCCCTTCAT TGCCGTGGGTGGAATCA	183 bp	[62]

**Table 2:** Particle size distribution metrics, specific surface area (SSA), and loading efficiency (LE%) of chitosan nanoparticles (CHNPs) and GnRH_a_–loaded chitosan nanoparticles (GnRH_a_–CHNPs).

Parameters	CHNPs (Before GnRHa Loading)	GnRH_a_–CHNPs (After GnRHa Loading)
Percentile diameters		
*D*_10_ (nm)	40	66
*D*_50_ (nm)	65	105
*D*_90_ (nm)	171	273
Mean diameter (*D*_1,0_; nm)	94	151
Surface area/Sauter mean diameter (*D*_3,2_; nm)	616	993
Volume/De Brouckere mean diameter (*D*_4,3_; nm)	2519	4188
Span *	1.99	1.96
Specific surface area (SSA; m^2^/kg)	3613	2241
Loading efficiency (LE %)	ND	90.5

* Span calculated as [(*D*_90_ − *D*_10_)/*D*_50_]. ND: Not detected.

## Data Availability

Data are contained within the article.

## Abbreviations

The following abbreviations are used in this manuscript:

AI	Artificial insemination
AT	Ambient temperature
*BAX*	BCL2-associated X protein
*BCL2*	B-cell lymphoma 2
*BMP15*	Bone morphogenetic protein
CAT	Catalase
CH	Chitosan
CHNPs	Chitosan nanoparticles
CL	Corpus luteum
CV%	Coefficient of variation
*D*	Hydrodynamic particle size
DF	Dominant follicles
E_2_	Estradiol
ELISA	Enzyme-linked immunosorbent assay
FSH	Follicle-stimulating hormone
*FSHR*	Follicle-stimulating hormone receptor
*GAPDH*	Glyceraldehyde-3-phosphate dehydrogenase
GCs	Granulosa cells
*GDF9*	Growth differentiation factor 9
GnRH	Gonadotropin-releasing hormone
GnRH_a_	GnRH analog
GnRH_a_–CHNPs	GnRH-loaded chitosan nanoparticles
HPA	Hypothalamic–pituitary–adrenal axis
HPO	Hypothalamic–pituitary–ovarian axis
HS	Heat stress
IGF-1	Insulin-like growth factor-1
*IGF-1R*	Insulin-like growth factor-1 receptor
LE%	Loading efficiency
LH	Luteinizing hormone
*LHR*	Luteinizing hormone receptor
MDA	Malondialdehyde
MOVS	Modified Ovsynch protocol
NO	Nitric oxide
OBI	Oxidative balance index
P_4_	Progesterone
PGF_2α_	Prostaglandin F_2α_
PR	Pregnancy
RH	Relative humidity
ROS	Reactive oxygen species
SOD	Superoxide dismutase
SOVS	Standard Ovsynch protocol
SSA	Specific surface area
*STAR*	Steroidogenic acute regulatory protein
TAC	Total antioxidant capacity
TAI	Timed artificial insemination
TCs	Theca cells
THI	Temperature–humidity index
TPP	Sodium tripolyphosphate
